# Thrombophilic Risk Factors in Patients With Inflammatory Bowel Disease

**DOI:** 10.4021/gr2010.06.209w

**Published:** 2010-05-20

**Authors:** Ayten Yazici, Omer Senturk, Cem Aygun, Altay Celebi, Cigdem Caglayan, Sadettin Hulagu

**Affiliations:** a Kocaeli University Medical Faculty Department of Internal Medicine, Kocaeli, Turkey; b Kocaeli University Medical Faculty Department of Internal Medicine and Gastroenterology, Kocaeli, Turkey; c Kocaeli University Medical Faculty Department of Public Health, Kocaeli, Turkey

**Keywords:** Inflammatory bowel disease, Ulcerative colitis, Crohn’s disease, Thrombosis

## Abstract

**Background:**

Inflammatory bowel disease (IBD) patients have an increased risk for thromboembolism. The aim of this study was to assess the presence of thrombophilic risk factors in IBD patients and to assess the associations of these factors with disease activity.

**Methods:**

Forty-eight patients with IBD (24 ulcerative colitis, 24 Crohn’s disease) and 40 matched healthy control individuals were enrolled. In addition to routine biochemical analysis, fasting blood samples were studied for prothrombin time (PT), activated partial thromboplastin time (aPTT), fibrinogen, protein-C, protein-S, antithrombin III, factor VII, factor VIII, D-dimer, vitamin B_12_, folic acid and homocysteine.

**Results:**

Levels of erythrocyte sedimentation rate (ESR), C-reactive protein (CRP), fibrinogen, D-dimer and the number of platelets were significantly higher in patients with IBD. When compared to control group, in patients with Crohn’s disease serum homocystein levels were significantly higher (p = 0.025) while serum folic acid levels were significantly lower (p < 0.019). Levels of fibrinogen, D-dimer, protein C, factor VIII, total homocystein and the number of platelets were found to be significantly higher in Crohn’s disease patients who were in active period of the disease.

**Conclusions:**

Thrombophilic defects are multifactorial and might be frequently seen in IBD patients. They might contribute to thrombotic complications of this disease.

## Introduction

Inflammatory bowel disease (IBD) patients are at an increased risk for venous and arterial thromboembolism [1, 2]. Although the exact pathogenetic mechanisms are unclear, abnormalities regarding platelets [3, 4], coagulation cascade [5], and fibrinolysis [6, 7] have been previously reported. Hyperhomocysteinaemia is also considered as another independent risk factor for thromboembolism in IBD; it may occur due to folate and vitamin B_12_ deficiencies or secondary to malabsorbtion [8]. In different clinical studies, prevalance of thromboembolic complications was reported at rates ranging from 1% to 7% and in autopsy series it was shown to reach up to 39% [1, 2, 9-11]. Thrombotic complications were also suggested to be increased during the active periods of disease [12]. Additionally several factors found by standard thrombophilia evaluation studies, such as hospitalization, immobilization, malignancy and recent surgery, may also contribute to increased thromboembolic complications in patients with IBD.

Although a wide spectrum of hereditary or acquired causes of thromboembolism such as deficiencies of protein C, protein S, antithrombin III (AT III), Factor V Leiden mutation, prothrombin 20210 mutation, methylene tetrahydrofolate reductase (MTHFR) mutations, high homocystein levels and presence of antiphospholipid antibodies have been reported in IBD patients, still there is no consistent data about most of them [13, 14]. Identification of any of those possible risk factors for both acquired and hereditary causes of thromboembolism in IBD might facilitate the the early management of patients. This study was aimed to investigate the presence of thrombophilic risk factors in a group of IBD patients who did not experience a major thromboembolic event previously.

## Patients and Methods

The study was conducted in the Kocaeli University, Department of Gastroenterology. Totally 48 IBD patients (24 ulcerative colitis, UC and 24 Chron’s disease, CD) were enrolled. As control group, 40 age-matched individuals (24 female, 16 male; ages between 20-60, mean age: 41.52 ± 9.23) were included. Baseline demographical and clinical characteristics of patients included in the study are presented in Table 1. IBD patients answered a questionnaire assessing the presence of thromboembolism, pregnancy, hepatitis or liver failure, recent surgery, blood or blood products transfusion in last 4 weeks, heparin or warfarin treatment and history of malignant diseases. Any suspected thromboembolic event was evaluated by a colored Doppler ultrasound (Toshiba Eccocee, Japan) besides the clinical and the laboratory evaluations. Diagnostic criteria for colour-coded duplex sonography were visualisation of an intraluminal thrombus in a deep vein, lack of or incomplete compressibility, absence of spontaneous flow, and following distal manipulation.

**Table 1:** Baseline Clinica and Demographical Characteristics of Patients Included in the Study

	Crohn’s Disease	Ulcerative Colitis	Control
Number	24	24	40
Mean Age, years	38.08 ± 12.30	41.08 ± 11.24	41.52 ± 9.23
Sex (F/M)	16/8	13/11	24/16
Duration of illness (year)	3.83 ± 2.14	5.00 ± 2.02	-
Disease Activity			
Active	11	8	-
Remission	13	16	-

Ongoing treatment modalities for inflammatory bowel disease were different in UC and CD patients and they are summarized in Table 2. None of IBD patients used sulfasalazine which might disturb folic acid absorbtion. Control group patients had normal physical examination findings and normal laboratory tests, without any history of disease or drug use. Blood samples were collected after an overnight fasting. Informed consent was obtained from each patient and the study protocol was approved by the local Ethics Committee of Kocaeli University.

**Table 2:** Ongoing Treatment Modalities of IBD Patients Included in the Study

		Crohn’s Disease n (%)	Ulcerative Colitis n (%)
5-ASA	User	19 (79.2%)	22 (91.7%)
	Non-user	5 (20.8%)	2 (8.3%)
Prednisolone	User	1 (4.2%)	5 (20.8%)
	Non-user	23 (95.8%)	19 (79.2%)
Azothiopurine	User	5 (20.8%)	3 (12.5%)
	Non-user	19 (79.2%)	21 (87.5%)
Surgery	Minor surgery (without ileocecal resection)	6 (25%)	2 (8.3%)
	No surgery	18 (75.0%)	22 (91.7%)

Complete blood count was done by Cell Dyn 3700 autoanalyser. ESR was measured by sedi-system, CRP was measured automatically by nephelometric system. Serum concentrations of homocysteine were measured by the İmmunlite 2000 analyser using chemiluminicance method. D-dimer and protein S levels were measured by STA-liatest kits via immuno-turbidimetric way. AT III and protein C levels are measured by STA-liatest kits via calorimetry method. Fibrinogen and coagulation factors (Factor VII, Factor VIII ) were measured quantitavely by STA kits using STA compact analyser. Disease activity at the time of study was reassessed using Harvey-Bradshaw index (active > 4 points) or Seo clinical activity index (severe > 220, moderate between 150 - 220 or mild-remission < 150) for Crohn’s disease and ulcerative colitis respectively. For practical purposes UC patients were grouped as in remission when the disease activity was mild and as in active period when disease activity was moderate or severe.

Statistical analysis was done with SPSS 11.5 for Windows. All results were expressed as mean ± SEM. Biochemical parameters of groups were compared by using nonparametric and parametric tests (Kruskal Wallis test or ANOVA) accordingly.

## Results

In IBD group, one patient was examined by doppler ultrasound due to suspect thromboembolism but the result was negative. Other patients in IBD group had no evidence of venous thrombosis of the leg or arm, pulmonary embolism, deep venous thrombosis, thrombosis of the mesenteric vein or thrombosis of any other location. Biochemical tests for thrombophilic parameters of IBD patients and control group were summarized in Table 3. Mean levels of CRP, ESR, fibrinogen, homocysteine, folic acid, D-dimer and the number of platelets were found to be significantly different in patients with inflammatory bowel disease although results of coagulation cascade tests did not show any significant difference.

**Table 3:** Plasma and Serum Thrombophilic Parameters of IBD Patients and Control Group

	Crohn’s Disease (n = 24)	Ulcerative Colitis(n = 24)	Control (n = 40)
CRP (mg/dl)	0.83 ± 1.40**	0.49 ± 0.73*	0.14 ± 0.16
Platelets (x100) (K/uL)	300 ± 113**	292 ± 126 *	228 ± 57
ESR (mm/h)	25.08 ± 24.5***	17.42 ± 18.98*	8.10 ± 5.43
APTT (sn)	29.60 ± 1.83	29.62 ± 3.61	29.76 ± 2.17
PT (sn)	12.45 ± 0.64	12.85 ± 0.74	12.74 ± 1.00
INR	1.02 ± 0.40	1.03 ± 0.60	1.03 ± 0.80
Fibrinogen (g/L)	4.48 ± 1.45***	3.74 ± 0.75	3.25 ± 0.71
Homocystein (mmol/L)	11.10 ± 3.11*	10.31 ± 2.58	9.35 ± 2.92
Folic Acid (ng/ml)	7.51 ± 2.92^*^	7.91 ± 3.61	9.15 ± 4.26
Vitamin B_12_ (pg/ml)	347.46 ± 197.15**	315.58 ± 99.77	256.79 ± 83.64
D-Dimer (µg/ml)	0.52 ± 0.47**	0.43 ± 0.38	0.28 ± 0.90
Antithrombin III (%)	113.58 ± 11.53	115.83 ± 7.30	116.88 ± 11.61
Factor VII (%)	113.67 ± 23.87	105.38 ± 25.14	117.48 ± 35.03
Factor VIII (%)	138.08 ± 44.57	143.38 ± 33.12	127.40 ± 45.13
Protein S (%)	110.08 ± 24.77	101.00 ± 23.17	106.88 ± 20.45
Protein C (%)	109.96 ± 27.98	106.46 ± 20.01	112.03 ± 24.67

p < 0.05; **p < 0.01; *** p < 0.001, when compared to the control group

As a marker of inflammation, mean CRP level of CD group was significantly higher in IBD group than control group, but the difference between UC and control groups in terms of CRP levels was not significant. Mean ESR levels of both CD and UC patients were significantly higher than control group. For both CD and UC patients, platelet count results were significantly higher than those of control group. Fibrinogen, homocystein, Vitamin B_12_ and D-Dimer levels were significantly higher in CD patients when compared to control group although differences in terms of these parameters between UC and control group did not reach a statistical significance. Mean folic acid level of CD patients was found significantly lower than control group. PT, APTT, INR, antithrombin III, factor VII, factor VIII, protein S, protein C results of both IBD patients and control group were similar.

When disease activity was considered, CD patients with active disease had significantly higher levels of platelets, fibrinogen, homocystein, D-dimer, protein C and factor VIII than CD patients with remission (Table 4). UC patients with remission did not show any significant difference from UC patients with active disease.

**Table 4:** Comparison of Thrombophilic Parameters Between CD Patients With Active Disease

Clinical Activity	Remission (n = 13)	Active Disease (n = 11)	*P*
Age (year)	39.63 ± 12.53	35.00 ± 12.04	0.397
Disease duration (year)	3.25 ± 1.84	5.00 ± 3.78	0.205
CRP (mg/dl)	0.46 ± 0.64	1.57 ± 2.14	0.067
Platelets (x1000) (K/uL)	273 ± 90	355 ± 139	0.033^*^
ESR (mm/h)	22.19 ± 19.60	26.88 ± 33.78	0.762
APTT (s)	30.10 ± 1.61	28.60 ± 1.96	0.058
PT (s)	12.53 ± 0.62	12.29 ± 0.71	0.407
INR	1.02 ± 0.05	1.01 ± 0.02	0.865
Fibrinojen (g/L)	4.13 ± 0.91	5.22 ± 2.07	0.043^*^
Homocystein (mmol/L)	9.56 ± 2.44	12.39 ± 2.67	0.028^*^
Folic Acid (ng/ml)	8.01 ± 3.61	7.81 ± 2.92	0.845
Vitamin B_12_ (pg/ml)	347.46 ± 197.15	345.32 ± 180	0.624
D-Dimer (µg/ml)	0.46 ± 0.42	0.74 ± 0.57	0.041^*^
Antithrombin III (%)	112.69 ± 12.04	115.38 ± 10.97	0.601
Factor VII (%)	113.75 ± 26.52	113.50 ± 19.12	0.981
Factor VIII (%)	122.06 ± 41.40	162.13 ± 43.17	0.0091^**^
Protein S (%)	111.88 ± 25.96	106.50 ± 23.46	0.627
Protein C (%)	106.25 ± 24.80	123.38 ± 34.06	0.037^*^

*p < 0.05; **p < 0.01

## Discussion

During the course of inflammatory bowel disease thromboembolic complications may occur. Several studies have given various prevalence rates as high as 39% seen in autopsy series [9-11]. However, although thromboembolic events are frequently found to be associated with IBD, the exact mechanism leading to such a hypercoagulable state among these patients is not clear yet. Data in the present study showed that multiple important thrombophilic defects are common in patients with IBD and they might have a role in increased thromboembolic complications. Additionally with this study we were able to show that even in IBD patients who experienced no major thromboembolic events, various thrombophilic risk factors might also occur.

Thrombophilic risk factors in IBD have been suggested to be related to multiple changes including inflammatory cytokines, inflammation, abnormal protein metabolism and therapeutic agents such as corticosteroids. Frequency of thromboembolic events was shown to increase with the increased disease activity. Pancolitis was also shown to be related to increased frequency of thromboembolic events [15, 16].

The most frequent thrombophilic change in IBD could be considered as thrombocytosis [17-19]. Thrombocytosis and enhanced thrombocyte functions increases the risk of thromboembolism [20-22]. Especially active period of IBD is shown to be associated with increased thrombocyte counts and with increased thrombocyte activity which can be shown by increased thromboxane B_2_ and β thromboglobulin levels [3]. Also thrombocyte aggregation is found to be enhanced in IBD [23]. Accordingly in the present study IBD group including both CD and UC patients showed significantly increased thrombocyte numbers when compared to healthy control individuals. Additionally when the activity of IBD was considered, patients with active disease had even more thrombocytes than patients with inactive IBD, although the difference between active UC and UC with remission did not reach a statistical significance.

As a sensitive marker of systemic inflammation, ESR and CRP are frequently increased in IBD [17, 24, 25]. Additionally both CRP and ESR is reported to be related to the activity of disease [26]. In the present study CRP and ESR levels of IBD patients were found to be significantly higher than control group. When disease activity of IBD patients was considered, both CRP and ESR levels did not show a significant difference between active and remission periods although they were both higher during the active period of disease. When the IBD patients were evaluated as ulcerative colitis and Chron’s disease seperately, CD group showed higher levels of CRP and ESR. The difference probably was due to higher systemic inflammatory activity seen among active CD patients.

D-dimer is a fibrin degradation product and used for the determination of intravascular thrombogenesis and fibrin cyclus. D-dimer is also a marker of inflammation and it is an acute phase reactant [27]. Intravascular thrombus and endothelial dysfunction could be seen in IBD and for this reason D-dimer might be increased, especially during the active period of disease while it could also be increased during the remission phase [28-30]. In our study D-dimer levels were increased among patients with IBD and D-dimer levels of active CD patients were found significantly higher than those of CD patients with remission. If we consider the elevated levels of ESR and CRP among patients with IBD ın our study, increased D-dimer levels were in accordance with increased inflammation hypothesis. The reason for the increased d-dimer in IBD could also be explained by activation of coagulation pathway, secondary fibrinolysis and inflammatory changes [31].

Fibrinogen is an acute phase reactant and increased in IBD [17, 21]. In various studies conducted about fibrinogen it was shown that fibrinogen increased in both active and remission periods of disease [16, 29]. In our study fibrinogen levels of IBD patients revealed significantly higher results than control group and there was a positive correlation between fibrinogen levels and activity of disease. Although a study about fibrinogen levels in IBD suggested that activity of disease was not important and it was increased in both active and remission periods of IBD [19], most of other studies about fibrinogen levels, including ours, suggested a positive correlation between fibrinogen and increased activity of IBD. In IBD patients, increased fibrinogen levels were also found to be correlated with increased D-dimer levels [28].

Among the familial thromboembolism cases, 14% - 24% is found to be related to AT III, protein C or protein S deficiency [33]. In active periods of IBD, due to intestinal protein loss, decreased ATIII, protein C and protein S levels might be expected [34]. While some studies among IBD patients demonstrated decreased protein C or protein S levels than normal population [15, 18, 33], some others showed no significant difference [17, 20, 30]. Protein S is 60% bound to plasma proteins and unbound protein S constitutes the active form. Studies measuring the total or unbound protein S levels may lead to conflicting results. In a study measuring unbound fraction of protein S, lower levels were shown with respect to control group [23]. Lower protein C and protein S levels were also shown to be related to active period of IBD [23, 36], although there were opposite ideas that indicated no difference between active or remission periods [37]. In the present study serum total protein C and protein S levels of IBD patients did not significantly differ from control group and there was no significant difference between active and remission period of disease neither. Similar to protein C and protein S, AT III which is an important physiologic thrombin inhibitor was found to be decreased in İBD in some studies [15, 27, 31]. But some other studies showed no difference [17, 23, 30, 33]. In our study there was no difference between IBD patients and control group in terms of serum AT III levels either.

Coagulation factors were suggested to be increased in IBD patients as acute phase reactants [25]. Especially factor VII, VIII, XIII and von Willebrand factor levels were found to be increased during active IBD [25, 35]. In our study factor VII and factor VIII levels were shown to be similar in IBD patients and control group. There was no difference between active and remission periods of UC patients while CD patients with active disease had significantly higher levels of factor VIII than CD patients with remission; this was probably due to acute phase reactant activity of this factor with increased inflammation seen among CD cases.

Homocysteine is a reactive aminoacid which has toxic side effects on vascular endothelium; it stimulates tissue factor which mainly starts coagulation and in the end elevated levels form an important risk factor for thromboembolism [38]. Hyperhomocysteinemia might be due to hereditary enzyme deficiencies, decreased levels of folic acid or vitamin B_12_, reduced oral intake, decreased absorption, resections due to complications and increased need [39-43]. Decreased folic acid levels of CD patients in our study might be one of the causes of hyperhomocysteinemia in this patient group. Sulfasalazine as frequent drug used in IBD disturbs folic acid absorption but use of this drug was nil in our groups [41, 43]. Homocystein levels increase and vitamin B_12_ levels might decrease in patients whom terminal ileum resection was performed but this major surgery including terminal ileum resection was not present in our study patients either [42]. Folic acid, vitamin B_12_ treated patients showed decreased homocysteine levels [39]. For this reason to overcome the conflicting results that could be due to use of these vitamins, a questionnaire assessing the use of medication was used and vitamin use was considered as a criteria for exclusion in our study. Some studies showed increased homocysteine levels in active periods of IBD but only a small part of these patients (7.2%) showed folic acid deficiency [15, 40, 41, 44, 45]. In our study homocysteine levels of CD patients were significantly higher than control group while interestingly the difference between UC patients and control group did not reach a significance level. Moreover active CD patients showed higher homocysteine levels than inactive CD patients indicating an increased risk for this subset of IBD group.

In conclusion, thromboembolic events in IBD should be considered multifactorial and they are not infrequent. Increased ESR, CRP, platelets, fibrinogen, D-dimer, factor VIII and homocysteine levels are important risk factors in IBD and might be seen especially during the active period of CD. However, need for more prospective studies remains clear to explain the exact factors underlying increased thromboembolic events in IBD.
